# Assessment and Correlation of Salivary Ca, Mg, and pH in Smokers and Non-Smokers with Generalized Chronic Periodontitis

**DOI:** 10.3390/medicina59040765

**Published:** 2023-04-14

**Authors:** Saad Mohammad Alqahtani, Shankar T. Gokhale, Mohamed Fadul A. Elagib, Deepti Shrivastava, Raghavendra Reddy Nagate, Badar Awadh Mohammad Alshmrani, Abduaziz Mohammed Abdullah Alburade, Fares Mufreh Abdullah Alqahtani, Anil Kumar Nagarajappa, Valentino Natoli, Kumar Chandan Srivastava

**Affiliations:** 1 Department of Periodontics and Community Sciences (PCS), College of Dentistry, King Khalid University, Abha 62529, Saudi Arabia; 2 Department of Preventive dentistry, College of Dentistry, Jouf University, Sakaka 72345, Saudi Arabia; 3 Department of Oral Maxillofacial Surgery & Diagnostic Sciences, College of Dentistry, Jouf University, Sakaka 72345, Saudi Arabia; 4 Department of Dentistry, School of Biomedical and Health Sciences, European University of Madrid, 28670 Madrid, Spain; 5 Private Dental Practice, 72015 Fasano, Italy; 6 Department of Oral Medicine and radiology, Saveetha Dental College, Saveetha Institute of Medical and Technical Sciences, Saveetha University, Chennai 602105, India

**Keywords:** periodontology, periodontitis, smokers, salivary calcium, probing pocket depth, salivary biomarkers, salivary magnesium, salivary pH, clinical dentistry

## Abstract

*Background and Objectives*: Diagnostic evaluation with the aid of biomarkers has reached newer heights to assess disease activity. Salivary calcium, magnesium, and pH are one of the biochemical parameters which can be helpful in assessing the progression of periodontal disease. Smokers are at topnotch threat for having oral diseases, predominantly periodontal diseases. The aim of this study was to assess the salivary calcium, magnesium, and pH levels in smokers compared with non-smokers with chronic periodontitis. *Materials and Methods*: The current study was conducted on 210 individuals affected with generalized chronic periodontitis, with the age group between 25 and 55 years. Based on their smoking habit, an equal number of patients were categorized into two groups; namely, group I consisted of non-smokers and group II consisted of smokers. The clinical parameters that were measured included Plaque Index (PI), Gingival Index (GI), Probing Pocket Depth (PPD), and Clinical Attachment Loss (CAL). The biochemical variables that were evaluated in the current study included salivary calcium, magnesium, and pH using an AVL9180 electrolyte analyzer (Roche, Germany). The gathered data were analyzed with an unpaired t test was using SPSS 20.0. *Results*: A statistically significant higher PPD (*p* < 0.01), CAL (*p* < 0.05), and salivary calcium levels (*p* < 0.001) were observed in the smokers’ compared with their non-smoking counterparts. Among the biochemical parameters, calcium showed a significantly (*p* < 0.001) higher level in smokers (5.79 ± 1.76) in contrast to non-smokers (3.87 ± 1.03). Additionally, a significant negative correlation (*p* < 0.05) between calcium and PPD was observed in non-smokers, whereas a non-significant inverse relation (*p* > 0.05) was seen in smokers. *Conclusions*: The present study indicates that the salivary calcium level can be a potential biochemical parameter to assess the progression of periodontal disease in smokers and non-smokers. Within the limitations of the current study, the salivary biomarkers appear to have an essential role in the identification and indication of the status of periodontal diseases.

## 1. Introduction

The metabolic profiles of human biofluids have been used for a long time to evaluate and differentiate an individual’s condition in terms of health or disease. Fluctuations have been observed in the volume and compositions of these fluids by virtue of a change in activity, drug usage, nutrition, or disease progression [1].

Oral cavities possess two prominent fluids including gingival crevicular fluid (GCF) and saliva. GCF, being in the closest proximity to gingival tissues, exhibits great potential in detecting periodontal disease and differentiating it from a healthy state [2]. Saliva has an indispensable role in various biological activities in the oral cavity and plays a pivotal role in its defense mechanism [3]. The whole saliva is a combination of fluids consisting of secretions from the major and minor salivary glands; gingival crevicular fluids; and oral mucosa transudate [4]. Thus, saliva is loaded with a variety of molecules and trace elements which make it a promising disease biomarker. Furthermore, it is easy to collect and store, as well as being easily resampled [5]. 

Ionomics is the study of the ionome, which is defined as a “mineral nutrient and trace element composition of an organism representing the inorganic component of the cellular and organ systems.” In recent years, salivary ionomes have emerged as a promising biomarker and thus have been projected as a vital diagnostic means to observe oral and systemic diseases. As a medium for clinical diagnosis, salivary biomarkers have a number of benefits over serum, such as the non-invasive nature of sample collection and the cost-effective approach, especially when targeting a large population [6,7].

As the main constituent, water comprises 99% of saliva, whereas the remaining 1% is made up of organic and inorganic constituents. The predominant electrolytes present in saliva include calcium, magnesium, potassium, sodium chloride, bicarbonate, and phosphate [8]. Salivary calcium has a close affinity for plaque formation that eventually influences the calculus formation. Since plaque and calculus are considered the main culprit in the etiopathogenesis of periodontal disease [9], the presence of an increased amount of calcium in saliva is known to influence plaque formation and its maturation. It has been observed that periodontally healthy participants with no marginal alveolar bone loss have a lesser potential for plaque and calculus mineralization in contrast to the patients who have been previously treated for periodontitis [10,11,12,13]. Magnesium is a known physical antagonist to calcium; however, the exact functional reciprocation in periodontitis or other risk factors associated with periodontitis, such as smoking, have not been explored. Nevertheless, there are a few studies that have shown the association of magnesium with periodontitis [14], and calcium and magnesium with periodontitis [15,16].

According to various cross-sectional and longitudinal studies, smoking is a significant risk factor for the development of periodontal disease [17,18]. Epidemiological as well as clinical studies are in alignment with the detrimental effects of smoking on periodontal tissues and, eventually, in the progression of periodontal disease that manifests as alveolar bone loss, increased probing depth, and tooth loss [19]. Additionally, it has been observed that smokers have poor oral hygiene and increased supragingival calculus formation [20]. It is well-documented that smoking induces a significant increase in the salivary flow rate as a spontaneous reflex action, which may explain the observation of increased calculus in smokers [21]. According to other research, smoking improves the mineralizing potential of saliva thus facilitating calculus formation [18].

Several studies have reported that patients with reduced bone mineral density, heavy smokers, and women in their menopausal ages have greater salivary calcium levels than age-matched peers [8,22,23]. The normal range of salivary calcium is 0.5–2.7 mmol/L [24]. In smokers, a higher level of salivary calcium is produced, which is linked to more bone loss and, accordingly, lower bone mineral density compared with non-smokers [25,26]. Salivary pH is normally between 6.2 and 7.6, with 6.7 being the average [25]. However, the pH of the oral cavity does not dip below 6.3 during rest and it is kept near neutral (6.7–7.3) by saliva [27]. Since smokers have a higher oral pH than non-smokers, there is more room for this pH to remove calcium and deposit it on teeth, perhaps resulting in high amounts of salivary calcium [28].

There are a few studies that have stated the role of calcium, magnesium, and pH in the progression of periodontal disease [1,11,12,17]. However, there is a lack of data about the appraisal and evaluation of salivary calcium and magnesium levels in smokers and non-smokers with chronic periodontitis. Hence, the present study aims to evaluate the effect of salivary calcium and magnesium in addition to the pH levels in smoking and non-smoking chronic periodontitis patients.

## 2. Materials and Methods

### 2.1. Study Characteristics 

A cross-sectional study was conducted at the College of Dentistry, King Khalid University, Abha, Saudi Arabia in the year 2019, after approval from the Institutional Ethical committee (SRC/ETH/2018-19/075). This study followed the protocol of the Declaration of Helsinki (1975) revised in 2002. 

### 2.2. Sample Characteristics 

A priori sample size calculation was performed using G* power software (Universität Düsseldorf: Psychologie—HHU) [29]. Considering t-test for comparing means of two independent study groups with equal allocation (Allocation ratio N_2_/N = 1), an effect size (Cohen’s d value) of 0.5, and a confidence interval (1-β error) of 95% and 0.05 α, a total sample of 210 was calculated. With this sample size, the power of the study was estimated to be 0.95.

Based on the inclusion and exclusion criteria, a total of 210 chronic generalized periodontitis patients were recruited from outpatient department. Later, based on smoking status, an equal number of patients (105) were divided into the two study groups, namely Group I, consisting of non-smokers, and Group II, of smokers. The patients who smoked at least one cigarette per day in the last year were considered active smokers and were included in the study group [14]. After explaining the purpose of the study, informed consent was obtained from all the patients participating in the study. 

The patients included were in the age range from 25 to 55 years, with at least 20 permanent teeth. Patients who were clinically diagnosed with chronic periodontitis presented with an evident bone loss on radiographical assessment and with a Probing Pocket Depth (PPD) of ≥4 mm with a Clinical Attachment Loss (CAL) of ≥1 mm. Patients who gave a history of periodontal therapy in last 6 months and had taken antibiotic coverage in last 3 months were excluded. Along with this, the patients on medications who were affected with a chronic disease which has influence on periodontal parameters were excluded from the study. Patients having xerostomia, either due to systemic or local conditions, were also excluded, as this could influence the periodontal conditions. 

### 2.3. Study Protocol and Clinical Parameters Measured in the Study 

A pre-designed data extraction sheet was used to collect information regarding demographic data and details such as medical history and oral hygiene practices. The clinical parameters including Loe and Silness Gingival Index (GI) [30], Bleeding on probing (BOP), Probing Pocket Depth (PPD), and Clinical Attachment Loss (CAL) were used for the assessment of the clinical condition. To lessen the bias, the measurements of all clinical parameters were documented and taken by a single examiner, who was initially calibrated. The intra-examiner reliability of the examiner for all the coding was 0.88, which was of good agreement. Plaque Index was measured after giving erythrosine in the form of a chewing tablet. BOP and CAL were assessed using a specific periodontal probe (UNC-15, Hu-friedy, Chicago, IL, USA). PPD was recorded from the gingival margin to the gingival sulcus base, while CAL was recorded from cemento-enamel junction (CEJ) to the base of the gingival sulcus. 

### 2.4. Collection of Salivary Sample and Its Laboratory Analysis 

A saliva sample was obtained after clinical recordings. A 2 mL of unstimulated whole saliva was collected by the “spitting method” as described by Navazesh M. (1993) [31]. To correspond to the circadian rhythm, salivary samples were collected 2 h after the last meal, after rinsing with water for 5 min. Patient was instructed to spit the saliva gathered in the floor of the mouth into the collecting unit. To avoid time-related alteration in pH of saliva, it was collected immediately. The samples were then sent to the laboratory within 24 h, with temperatures maintained at 2 to 4 degrees Celsius. Salivary pH was measured using pH litmus test paper. AVL9180 electrolyte analyzer (Roche, Germany) was used for measuring calcium and magnesium ions. 

### 2.5. Data Analysis 

The data collected were analyzed using statistical package of social sciences (SPSS) 20.0 version (IBM; Chicago, IL, USA). The gathered data were initially checked for normality with Kolmogorov–Smirnov test and visualization methods including histogram and Q-Q plots. All the variables tested in the current study were found to be normally distributed (*p* > 0.05). Results were expressed as means and standard deviation. Based on the normality distribution of the data, parametric test–Unpaired t test was used to compare the clinical and biochemical parameters between the study groups. Correlation among the clinical and biochemical were analyzed using Pearson’s and Spearman correlations for parametric and categorial type of variable, respectively.

## 3. Results

### 3.1. Sample Characteristics

There was no significant difference (*p* > 0.05) in the age and gender distribution between the groups, with smokers having a mean age of 42.1 ± 2.3 years and non-smokers having a mean age of 45.8 ± 3.4 years (Table 1).

### 3.2. Comparative Analysis of Clinical Parameters between the Study Groups

There is a significantly higher PPD (*p* < 0.05) and CAL (*p* < 0.01) in the smoker group compared with the non-smoker patients. However, non-significant (*p* > 0.05) differences in PI and GI were observed in the study group when compared with the control group patients. (Table 2).

### 3.3. Comparative Analysis of Biochemical Parameters between the Study Groups

Among the biochemical parameters, significantly (*p* < 0.001) raised calcium levels (5.79 ± 1.76 mmol/L) were observed in smokers when compared with non-smokers (3.86 ± 1.03 mmol/L). However, magnesium did not show any difference between the groups. (Table 3)

### 3.4. Correlational Analysis of Biochemical and Clinical Parameter in the Study Groups

Depending on the type of variable (parameteric Vs. non-parameteric), Pearson and Spearman correlation analysis was carried out for all variables in both study groups. 

The two crucial periodontal clinical indicators, namely PPD and CAL, showed a highly significant (*p* < 0.001) positive correlation in both study groups. Additionally, PPD and CAL were later analyzed with calcium and magnesium. Similarly, the gingival parameters, namely PI and GI, showed a significant (*p* < 0.001) positive correlation in Group I and a positive but non-significant (*p* > 0.05) correlation in Group II. These correlation results reaffirm the presentation of periodontal diseases. 

Another key parameter, the pH of the saliva, showed a significant (*p* < 0.05) negative correlation with magnesium in Group I and a significant (*p* < 0.05) negative correlation with calcium in Group II.

**Figure 1:**
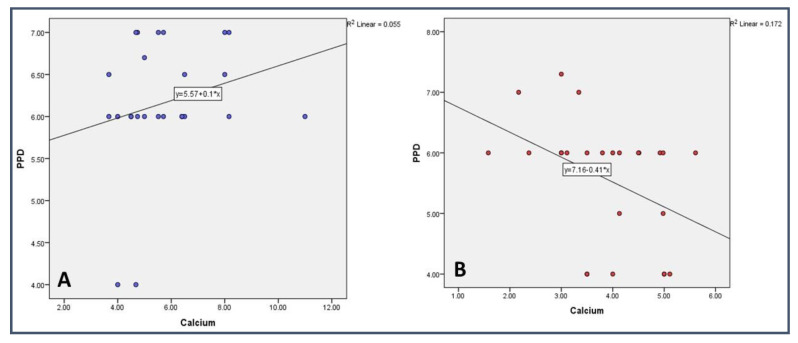
Correlational analysis of Calcium with Periodontal Probing Depth in (**A**) Smokers and (**B**) Non-Smokers.

**Figure 2:**
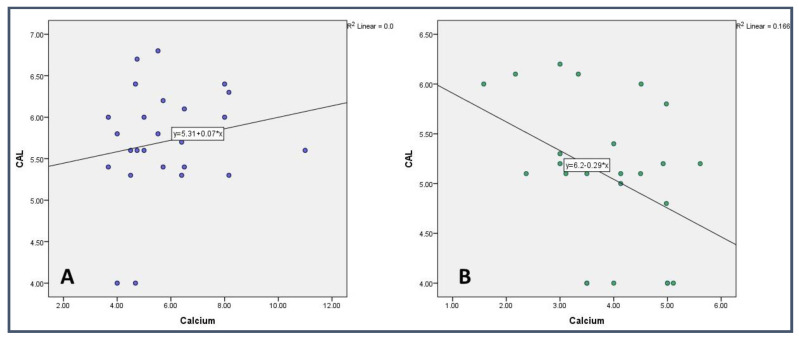
Correlational analysis of Calcium with Clinical Attachment Loss in (**A**) Smokers and (**B**) Non-Smokers.

The correlation analysis between salivary calcium and periodontal clinical parameters such as CAL and PPD was carried out in each study group. In the control group (Non-smoker), a significant (*p* < 0.05) negative correlation was found between calcium and PPD and CAL. However, a non-significant (*p* > 0.05) positive correlation was seen between the parameters in the study group (Table 4 and Table 5) (Figure 1 and Figure 2).

## 4. Discussion

With the advancement of research, the metabolites profiling of a biological system has been commonly utilized to provide insight into the normal and disconcert metabolic processes [32]. Salivary metabolites can act as a biomarker to understand the complex biochemical interaction of host and bacteria in periodontal diseases [33,34,35]. It has been proven with various studies that tobacco smoke can alter the biochemical composition, and subsequently the function, of saliva [36,37].

Salivary Ca and Mg can be considered imperative in periodontal health concerning their influence on plaque mineralization. Magnesium may play an important role in preventing periodontal disease as it has a unique ability to reduce inflammation caused by bacterial toxins [18]. A group of studies reported that reduced magnesium concentrations are associated with an enhanced inflammatory response to bacterial challenges, thus promoting periodontitis [15,38]. Conversely, Manea et al. reported that salivary Mg concentrations were significantly higher in the periodontitis group compared with the controls. In another study, it was observed that salivary Mg concentrations were higher in smokers with periodontitis than in non-smokers who were also affected by periodontitis [38]. Although in the present study reduced Mg levels were reported in the smoker group compared with the non-smokers, the difference was non-significant. Similarly, Mg levels showed a non-significant negative correlation with PPD and CAL in both groups. A similar correlation was reported between Mg and periodontal parameters in the study conducted by Erdemir EO et al. [39]. 

Smokers have been classified as light smokers who smoke one–ten cigarettes a day; moderate smokers who smoke eleven–twenty cigarettes a day; and heavy smokers who smoke more than twenty cigarettes a day [14,40]. Smoking is thought to increase salivary Ca levels independently by reducing skeletal bone density [40]. The literature highlights the increased Ca levels in periodontitis patients [31,35]. However, it is important to note that dietary calcium intake and overall calcium turnover can influence salivary calcium levels [41]. In addition, the continuous exposure of taste receptors to tobacco products such as nicotine probably affects salivary flow rate [42], salivary reflex, and also salivary Ca levels [8]. Smokers have fairly eminent levels of salivary calcium, which is allied with a greater degree of bone loss and lower bone mineral density than non-smokers. The present study showed significantly elevated Ca levels in smokers when compared with non-smokers. A study by Megha Varghese et al. and Kolte et al. reported analogous findings in a sample of periodontitis patients, with salivary calcium ranging higher in the smoker group than in non-smokers [43,44]. Gupta VV et al. also observed concordant findings in their study, wherein calcium level was increased in smokers diagnosed with aggressive periodontitis [45]. This was contradictory to the study of Ivana Sutej et al. and Shashikanth et al. who found no difference in calcium levels between smokers and non-smokers [28,46]. A study conducted by Zuabi et al. observed a reduction in calcium levels post treatment of periodonitis patients [47]. A higher calcium level was observed in the stimulated saliva of smokers in studies conducted by Sevon et al. [48] and Mc Gregor et al. [25]. According to sevon et al., the decreased bone mineral density, a side effect of smoking, could be a reason for high salivary calcium [48]. 

The normal salivary pH ranges from 6.2 to 7.6. The buffering capacities of saliva and salivary flow both have an impact on salivary pH [37]. It was observed in one of the studies that salivary pH was lower in periodontitis patients compared with healthy controls. There was no significant difference in pH readings amongst the groups, although it was more acidic in the smokers’ group [27]. Similar findings were observed in a study conducted by Kumar et al. which found a lower pH in smokers with periodontitis [49]. In contrast, the study of Gupta VV et al. showed a significant increase in pH levels in smokers against healthy controls [45] which could be due to the different technique adopted for the collection of saliva. However, the present study did not establish any significant difference in pH between the groups. The current study utilized the unstimulated saliva collection procedure as it bathes the oral cavity predominantly and moistens the oral cavity round the clock. Furthermore, it also represents the pooled sub-gingival plaque sample [50]; whereas, in other studies, stimulated saliva was collected [45]. Additionally, in a study conducted to evaluate the pH of smokers with traditional smoking and e-cigarette smoking and non-smokers, it was found that the traditional smokers and e-cigarette smokers had a lower pH than non-smokers [37]. 

On comparing the clinical parameters, such as PPD and CAL, smokers had more PPD and CAL compared with non-smokers with periodontitis. A similar observation was noticed by Haffajee AD et al. [51], Shashikanth H et al. [46], and Velidandla S et al. [52]. On comparing the Plaque Index, no difference was found between the groups. A similar observation was noticed in a study conducted by Sreedevi et al. [53]. When the Gingival Index was compared between the groups, no statistically significant difference was found. A similar finding was reported in other studies [54]. However, this result is contradictory to another study conducted by Zuabi et al. [47]. In a study conducted by Erdemir et al., they found that, in smokers, there was a positive correlation between the levels of Ca, Mg, and CAL. Whereas, in the non-smoker group, there was a negative correlation between the mean level of sodium and the Plaque Index (*p* < 0.05) [39]. In our study, we found that, in the non-smokers, there was a significant negative correlation between calcium and PPD and CAL. However, a non-significant (*p* > 0.05) positive correlation was seen between the parameters in the smokers’ group. The difference in the study could be because of the assessment method as in the previous study inductively coupled plasma–atomic emission spectrophotometry was used. However, in the present study, an AVL9180 electrolyte analyzer was used for assessment.

### Limitations and Future Directions

Within the limitations of this study, confounding factors such as the presence of calcium in the diet and differences in age were not addressed in this study. Therefore, longitudinal studies are recommended for establishing the causal relationship between the parameters. This will also aid the scientific society in winding up the judgment against the role of saliva in the initiation and progression of periodontal disease.

## 5. Conclusions

Among all the constituents of saliva, salivary calcium is one of the most extensively studied potential markers for the identification of periodontal diseases. The present study draws attention towards the specific risk factors that could influence the pathogenesis of periodontal disease, amid which smoking is a prompt factor. Smoking also serves as an indirect biomarker for periodontal lesion predilection. The results of the current study indicate that smokers have significantly higher PPD, CAL, and calcium than their non-smoking counterparts. Importantly, salivary calcium was found to be elevated in smokers with chronic generalized periodontitis, thus the attempts to signify that calcium levels in saliva act as both a risk factor and imminent biochemical marker for the assessment of periodontal lesions.

## Figures and Tables

**Table 1:** Sample Characteristics.

Variable	Categories	Study Group		*p* Value
		Group I (Non-Smoker) *n* = 105	Group II (Smoker) *n* = 105	
Age (Mean ± SD)		45.80 ± 3.46	42.08 ± 6.19	0.573
Gender ^†^	Male	57 (54)	59 (56)	0.62
	Female	48 (46)	46 (44)	

Note: ^†^—results expressed in Number (%); SD—Standard Deviation.

**Table 2:** Comparative analysis of clinical parameters among the study group.

Clinical Parameter	Group I	Group II	*p* Value
Plaque Index	1.71 ± 0.48	1.51 ± 0.34	0.109
Gingival Index	1.55 ± 0.38	1.67 ± 0.29	0.222
Periodontal Probing Depth	5.57 ± 1.02	6.16 ± 0.77	0.025 *
Clinical Attachment Level	5.08 ± 0.73	5.70 ± 0.67	0.003 **

Note: results expressed in Mean ± Standard Deviation; * *p* < 0.05; ** *p* < 0.01.

**Table 3:** Comparative analysis of biochemical parameters among the study group.

Parameter	Group I	Group II	*p* Value
pH	6.44 ± 0.86	6.80 ± 0.91	0.160
Calcium	3.86 ± 1.03	5.79 ± 1.76	0.000 ***
Magnesium	0.54 ± 0.18	0.49 ± 0.24	0.413

Note: results expressed in Mean ± Standard Deviation; *** *p* < 0.001.

**Table 4:** Correlation analysis of parameters in Group I.

	Ca	Mg	pH	PI	GI	PPD	CAL	Age	Gender ^¶^
Ca	-	0.985 (0.004)	0.243 (0.237)	0.978 (−0.006)	0.994 (0.002)	0.039 * (−0.415)	0.043 * (−0.407)	0.415 (−0.171)	0.490 (−0.145)
Mg	0.985 (0.004)	-	0.037 * (−0.419)	0.306 (0.213)	0.671 (0.089)	0.855 (−0.038)	0.789 (−0.056)	0.176 (0.280)	0.853 (−0.039)
pH	0.243 (0.237)	0.037 * (−0.419)	-	0.105 (0.332)	0.068 (0.371)	0.423 (0.168)	0.370 (0.187)	0.616 (0.105)	0.688 (0.084)
PI	0.978 (−0.006)	0.306 (0.213)	0.105 (0.332)	-	0.000 *** (0.783)	0.521 (0.135)	0.570 (0.119)	0.677 (0.088)	0.739 (0.070)
GI	0.994 (0.002)	0.671 (0.089)	0.068 (0.371)	0.000 *** (0.783)	-	0.902 (0.026)	0.865 (−0.036)	0.064 (0.376)	0.711 (0.078)
PPD	0.039 * (−0.415)	0.855 (−0.038)	0.423 (0.168)	0.521 (0.135)	0.902 (0.026)	-	0.000 *** (0.937)	0.834 (0.044)	0.725 (0.074)
CAL	0.043 * (−0.407)	0.789 (−0.056)	0.370 (0.187)	0.570 (0.119)	0.865 (−0.036)	0.000 *** (0.937)	-	0.791 (−0.056)	0.669 (0.090)
Age	0.415 (−0.171)	0.176 (0.280)	0.616 (0.105)	0.677 (0.088)	0.064 (0.376)	0.834 (0.044)	0.791 (−0.056)	-	0.811 (0.050)
Gender	0.490 (−0.145)	0.853 (−0.039)	0.688 (0.084)	0.739 (0.070)	0.711 (0.078)	0.725 (0.074)	0.669 (0.090)	0.811 (0.050)	-

Note: results are expressed as *p* value (correlation coefficient); * *p* < 0.05; *** *p* < 0.001; ^¶^—Spearman Correlation; Ca—Calcium; Mg—Magnesium; PI—Plaque Index; GI—Gingival Index; PPD—Probing Pocket Depth; CAL—Clinical Attachment Loss.

**Table 5:** Correlation analysis of parameters in Group II.

	Ca	Mg	pH	PI	GI	PPD	CAL	Age	Gender ^¶^
Ca	-	0.614 (−0.106)	0.003 ** (−0.572)	0.403 (0.175)	0.343 (0.198)	0.260 (0.234)	0.385 (0.182)	0.058 (0.385)	0.692 (0.083)
Mg	0.614 (−0.106)	-	0.488 (−0.145)	0.580 (0.116)	0.426 (−0.167)	0.667 (−0.090)	0.693 (−0.083)	0.473 (0.151)	0.145 (0.300)
pH	0.003 ** (−0.572)	0.488 (−0.145)	-	0.089 (0.347)	0.201 (0.265)	0.850 (−0.040)	0.976 (−0.006)	0.772 (−0.061)	0.286 (−0.222)
PI	0.403 (0.175)	0.580 (0.116)	0.089 (0.347)	-	0.068 (0.371)	0.444 (0.160)	0.327 (0.204)	0.270 (0.229)	0.453 (0.157)
GI	0.343 (0.198)	0.426 (−0.167)	0.201 (0.265)	0.068 (0.371)	-	0.632 (0.101)	0.492 (0.144)	0.033 * (0.427)	0.689 (0.084)
PPD	0.260 (0.234)	0.667 (−0.090)	0.850 (−0.040)	0.444 (0.160)	0.632 (0.101)	-	0.000 *** (0.963)	0.386 (0.181)	0.098 (−0.338)
CAL	0.385 (0.182)	0.693 (−0.083)	0.976 (−0.006)	0.327 (0.204)	0.492 (0.144)	0.000 *** (0.963)	-	0.388 (0.180)	0.054 (−0.390)
Age	0.058 (0.385)	0.473 (0.151)	0.772 (−0.061)	0.270 (0.229)	0.033 * (0.427)	0.386 (0.181)	0.388 (0.180)	-	0.222 (0.253)
Gender ^¶^	0.692 (0.083)	0.145 (0.300)	0.286 (−0.222)	0.453 (0.157)	0.689 (0.084)	0.098 (−0.338)	0.054 (−0.390)	0.222 (0.253)	-

Note: results are expressed as p value (correlation coefficient); ^¶^—Spearman Correlation; * *p* < 0.05; ** *p* < 0.01; *** *p* < 0.001; Ca—Calcium; Mg—Magnesium; PI—Plaque Index; GI—Gingival Index; PPD—Probing Pocket Depth; CAL—Clinical Attachment Loss.

## Data Availability

The data are available on a reasonable request from the corresponding author.

## References

[1] Velsko I.M., Overmyer K.A., Speller C., Klaus L., Collins M.J., Loe L., Frantz L.A., Sankaranarayanan K., Lewis C.M., Martinez J.B. (2017). The dental calculus metabolome in modern and historic samples. Metabolomics.

[2] Bibi T., Khurshid Z., Rehman A., Imran E., Srivastava K., Shrivastava D. (2021). Gingival Crevicular Fluid (GCF): A Diagnostic Tool for the Detection of Periodontal Health and Diseases. Molecules.

[3] Dawes C., Pedersen A.L., Villa A., Ekström J., Proctor G.B., Vissink A., Aframian D., McGowan R., Aliko A., Narayana N. (2015). The functions of human saliva: A review sponsored by the World Workshop on Oral Medicine VI. Arch. Oral. Biol..

[4] Castagnola M.P.M.P., Picciotti P.M., Messana I., Fanali C., Fiorita A., Cabras T., Calo L., Pisano E., Passali G.C., Iavarone F. (2011). Potential application of human saliva as diagnostic fluid. Acta Otorhinolaryngol. Ital..

[5] Dame Z.T., Aziat F., Mandal R., Krishnamurthy R., Bouatra S., Borzouie S., Guo A.C., Sajed T., Deng L., Lin H. (2015). The human saliva metabolome. Metabolomics.

[6] Sonalee S., Manpreet K. (2012). A study of analytical indicators of saliva. Ann. Essences Dent..

[7] Al Kawas S., Rahim Z.H., Ferguson D.B. (2012). Potential uses of human salivary protein and peptide analysis in the diagnosis of disease. Arch. Oral. Biol..

[8] Tjahajawati S., Rafisa A., Lestari E.A. (2021). The Effect of Smoking on Salivary Calcium Levels, Calcium Intake, and Bleeding on Probing in Female. Int. J. Dent..

[9] Shrivastava D., Srivastava K.C., Ganji K.K., Alam M.K., Al Zoubi I., Sghaireen M.G. (2021). Quantitative Assessment of Gingival Inflammation in Patients Undergoing Nonsurgical Periodontal Therapy Using Photometric CIELab Analysis. BioMed Res. Int..

[10] Giannobile W.V., Beikler T., Kinney J.S., Ramseier C.A., Morelli T., Wong D.T. (2009). Saliva as a diagnostic tool for periodontal disease: Current state and future directions. Periodontol. 2000.

[11] Acharya A., Kharadi M.D., Dhavale R., Deshmukh V.L., Sontakke A.N. (2011). High salivary calcium level associated with periodontal disease in Indian subjects—A pilot study. Oral. Health Prev. Dent..

[12] Sudhir S. (2010). Quantitative evaluation of salivary calcium, phosphorous, protein and Ph in health and diseased periodontium. Ann. Essences Dent..

[13] Shrivastava D., Srivastava K.C., Dayakara J.K., Sghaireen M.G., Gudipaneni R.K., Al-Johani K., Baig M.N., Khurshid Z. (2020). BactericidalActivity of Crevicular Polymorphonuclear Neutrophils in Chronic Periodontitis Patients and Healthy Subjects under the Influence of Areca Nut Extract: An In Vitro Study. Appl. Sci..

[14] Wijaya T.K., Susanto A., Hendiani I. (2021). Comparison of gingival health status and salivary magnesium levels in smokers and nonsmokers. Sci. Dent. J..

[15] Rajesh K.S., Zareena S.H., Kumar M.A. (2015). Assessment of salivary calcium, phosphate, magnesium, pH, and flow rate in healthy subjects, periodontitis, and dental caries. Contemp. Clin. Dent..

[16] Patel R.M., Varma S., SuRaGiMath G., ZoPe S. (2016). Estimation and comparison of salivary calcium, phosphorous, alkaline phosphatase and pH levels in periodontal health and disease: A cross-sectional biochemical study. J. Clin. Diagn. Res. JCDR.

[17] Sewón L., Söderling E., Karjalainen S. (1990). Comparative study on mineralization related intraoral parameters in periodontitis affected and periodontitis-free adults. Scand. J. Dent. Res..

[18] Calsina G., Ramón J.M., Echeverría J.J. (2002). Effects of smoking on periodontal tissues. J. Clin. Periodontol..

[19] Nociti F.H., Casati M.Z., Duarte P.M. (2015). Current perspective of the impact of smoking on the progression and treatment of periodontitis. Periodontol. 2000.

[20] Bergstrom J. (1999). Tobacco smoking and supragingival dental calculus. J. Clin. Periodontol..

[21] Petrovic M., Kesic L., Obradovic R., Savic Z., Mihailovic D., Obradovic I., Avdic-Saracevic M., Janjic-Trickovic O., Janjic M. (2013). Comparative analysis of smoking influence on periodontal tissue in subjects with periodontal disease. Mater. Sociomed..

[22] Wasti A., Wasti J., Singh R. (2020). Estimation of salivary calcium level as a screening tool for the osteoporosis in the post-menopausal women: A prospective study. Indian J. Dent. Res..

[23] Saha M.K., Agrawal P., Saha S.G., Vishwanathan V., Pathak V., Saiprasad S.V., Dhariwal P., Dave M. (2017). Evaluation of correlation between salivary calcium, alkaline phosphatase and osteoporosis-a prospective, comparative and observational study. J. Clin. Diagn. Res. JCDR.

[24] Rockenbach M.I., Marinho S.A., Veeck E.B., Lindemann L., Shinkai R.S. (2006). Salivary flow rate, pH, and concentrations of calcium, phosphate, and sIgA in Brazilian pregnant and non-pregnant women. Head Face Med..

[25] MacGregor I.D.M., Edgar W.M. (1986). Calcium and phosphate concentrations and precipitate formation in whole saliva from smokers and non-smokers. J. Periodontal Res..

[26] Sevon L.A., Laine M.A. (2008). Effect of age on flow rate, protein and electrolyte composition of stimulated whole saliva in healthy, non-smoking women. Open Dent. J..

[27] Baliga S., Muglikar S., Kale R. (2013). Salivary pH: A diagnostic biomarker. J. Indian Soc. Periodontol..

[28] Ivana S., Kristina P., Anica B., Krunoslav C., Kresimir B., Kata R.G. (2012). Salivary calcium concentration and periodontal health of young adults in relation to tobacco smoking. Oral. Health Prev. Dent..

[29] Faul F., Erdfelder E., Lang A.-G., Buchner A. (2007). G*Power 3: A flexible statistical power analysis program for the social, behavioral, and biomedical sciences. Behav. Res. Methods.

[30] Loe H. (1967). The Gingival Index, the Plaque Index and Retention Index systems. J. Periodontol..

[31] Navazesh M. (1993). Methods for collecting saliva. Ann. N. Y Acad. Sci..

[32] Clarke C.J., Haselden J.N. (2008). Metabolic profiling as a tool for understanding mechanisms of toxicity. Toxicol Pathol..

[33] Kuboniwa M., Sakanaka A., Hashino E., Bamba T., Fukusaki E., Amano A. (2016). Prediction of periodontal inflammation via metabolic profiling of saliva. J Dent Res..

[34] Gardner A., Carpenter G., So P.W. (2020). Salivary metabolomics: From diagnostic biomarker discovery to investigating biological function. Metabolites.

[35] Liebsch C., Pitchika V., Pink C., Samietz S., Kastenmüller G., Artati A., Suhre K., Adamski J., Nauck M., Völzke H. (2019). The saliva metabolome in association to oral health status. J. Dent. Res..

[36] Macgregor I.D. (1988). Smoking, saliva and salivation. J. Dent..

[37] Cichońska D., Kusiak A., Kochańska B., Ochocińska J., Świetlik D. (2022). Influence of Electronic Cigarettes on Selected Physicochemical Properties of Saliva. Int. J. Environ. Res. Public. Health.

[38] Manea A., Nechifor M. (2014). Research on plasma and saliva levels of some bivalent cations in patients with chronic periodontitis (salivary cations in chronic periodontitis). Rev. Med. Chir. Soc. Med. Nat. Iasi.

[39] Erdemir E.O., Erdemir A. (2006). The detection of salivary minerals in smokers and non-smokers with chronic periodontitis by the inductively coupled plasma-atomic emission spectrophotometry technique. J. Periodontol..

[40] Sewon L.A., Karjalainen S.M., Sainio M., Seppä O. (1995). Calcium and other salivary factors in periodontitis-affected subjects prior to treatment. J. Clin. Periodontol..

[41] Najeeb S., Zafar M.S., Khurshid Z., Zohaib S., Almas K. (2016). The Role of Nutrition in Periodontal Health: An Update. Nutrients.

[42] Sewon L.A., Karjalainen S.M., Söderling E., Lapinlaimu H., Simell O. (1998). Association between salivary calcium and oral health. J. Clin. Periodontol.

[43] Varghese M., Hegde S., Kashyap R., Maiya A.K. (2015). Quantitative assessment of calcium profile in whole saliva from smokers and non-smokers with chronic periodontitis. J. Clin. Diagn. Res..

[44] Kolte A.P., Kolte R.A., Laddha R.K. (2012). Effect of smoking on salivary composition and periodontal status. J. Indian Soc. Periodontol..

[45] Gupta V.V., Chitkara N., Gupta H.V., Singh A., Gambhir R.S., Kaur H. (2016). Comparison ofsalivary calcium level and ph in patients with aggressive periodontitis and healthy individuals: A clinico-biochemical study. Oral. Health Dent. Manag..

[46] Shashikanth H., Raghavendra U., Naveena N., Rajesh K.S. (2016). Assessment of Salivary Composition in Smokers and Non Smokers with Chronic Periodontitis. J. Dent. Med. Sci..

[47] Zuabi O., Machtei E.E., Ben-Aryeh H., Ardekian L., Peled M., Laufer D. (1999). The effect of smoking and periodontal treatment on salivary composition in patients with established periodontitis. J. Periodontol..

[48] Sewon L., Laine M., Karjalanien S., Dorpguinskania A., Lentonen-Veromaa M. (2004). Salivary calcium concentration reflects skeletalosteoporotic changes in heavy smokers. Arch. Oral. Biol..

[49] Kumar C.N., Rao S.M., Jethlia A., Linganna C.S., Bhargava M., Palve D.H. (2021). Assessment of salivary thiocyanate levels and pHin the saliva of smokers and nonsmokers with chronic periodontitis—A comparative study. Indian J. Dent. Res..

[50] Rane M.V., Suragimath G., Varma S., Zope S.A., Ashwinirani S.R. (2017). Estimation and comparison of salivary calcium levels in healthy controls and patients with generalized gingivitis and chronic periodontitis. J. Oral. Res. Rev..

[51] Haffajee A.D., Socransky S.S. (2001). Relationship of smoking to attachment level profiles. J. Clin. Periodontol..

[52] Velidandla S., Bodduru R., Birra V., Jain Y., Valluri R., Ealla K.K. (2019). Distribution of periodontal pockets among smokers and Nonsmokers in patients with chronic periodontitis: A cross-sectional study. Cureus J. Med. Sci..

[53] Sreedevi M., Ramesh A., Dwarakanath C. (2012). Periodontal status in smokers and nonsmokers: A clinical, microbiological, and histopathological study. Int. J. Dent..

[54] Haber J., Wattles J., Crowley M., Mandell R. (1993). Evidence for cigarette smoking as a major risk factor for periodontitis. J. Periodontol..

